# What the Eyelid Can Tell You: The Unexpected Initial Presentation of De Novo Stage IV Breast Carcinoma

**DOI:** 10.1111/cup.70013

**Published:** 2025-12-04

**Authors:** Grace L. Casado, Eileen Xu, Maedot A. Haymete, Nicholas A. Ramey, Douglas J. Grider

**Affiliations:** ^1^ Virginia Tech Carilion School of Medicine Roanoke Virginia USA; ^2^ Oculofacial Plastic and Reconstructive Surgery Vistar Eye Center Roanoke Virginia USA; ^3^ Division of Dermatology, Department of Internal Medicine Virginia Tech Carilion School of Medicine Roanoke Virginia USA; ^4^ Dominion Pathology Associates Roanoke Virginia USA; ^5^ Department of Basic Science Education Virginia Tech Carilion School of Medicine Roanoke Virginia USA

**Keywords:** breast carcinoma, ductal adenocarcinoma, E‐cadherin, eyelid, lobular carcinoma, metastases, P120‐catenin

## Abstract

A 66‐year‐old female presented with seven months of progressive right upper eyelid (RUL) drooping and thickening of her right lower eyelid (RLL). MRI revealed soft tissue enhancement of the RUL and RLL pre‐septal planes without posterior extension. Biopsy revealed poorly cohesive carcinoma infiltrating in a linear architectural pattern with foci of signet ring cell forms. Positivity for mucicarmine, keratin CAM5.2, CK7, GATA3, BRST‐2, mammaglobin, and ER supported a metastatic breast carcinoma to the eyelid without a previously known primary site. E‐cadherin and p120‐catenin membranous staining was suggestive of a ductal breast carcinoma with lobular features as the initial presentation of de novo stage IV breast carcinoma. Subsequent follow‐up with oncology revealed a palpable right breast mass with associated lymphadenopathy. Estrogen receptor PET scan showed disease in the right breast, right axilla, left cervical nodes, calvarium, and orbit. Biopsy of the right breast lesion confirmed a carcinoma histopathologically and immunohistochemically identical to that found in the eyelid biopsy. This case's histopathological features of invasive ductal breast carcinoma masquerading as invasive lobular carcinoma exemplify the challenging complexity of mixed disease.

## 1 Introduction

The most common breast cancer subtype is invasive ductal carcinoma (IDC), comprising 73% of cases compared to 15% with invasive lobular carcinoma (ILC) [1]. Histologically, IDC features malignant ductal proliferation with stromal invasion [2]. ILC typically has a linear, single‐file, cord‐like pattern of dyscohesive cells [2]. 3%–5% of breast carcinomas are mixed IDC/ILC phenotypes, featuring a heterogenous appearance of both single cell infiltrative patterns and areas of cohesive cell growth and tubule formation [3].

Although generally seen later in the disease course, cutaneous metastasis of breast carcinoma occurs in 23.9% of cases [4], which most often occurs at the chest due to its close proximity [5]. Less frequently, metastases to the skin can be seen on the eyelid in 2.3% of breast cases [6]. Only an estimated 3% of cutaneous manifestations are the initial presentations that lead to the diagnosis of primary breast carcinoma [7]. Even fewer are eyelid metastases as the presenting sign of breast carcinoma, with only three other reports in the literature to date [8, 9, 10]. We report the fourth documented case of eyelid metastases as the initial presenting sign of an underlying breast carcinoma. Immunohistochemical studies in the three previous cases led to a straightforward diagnosis of two ILCs and one IDC. This case, however, is the first to report eyelid metastases as the initial presentation of a diagnostically challenging underlying breast carcinoma whereby IDC was masquerading as ILC. Described herein is a patient presenting with an eyelid lesion that subsequently led to the diagnosis of de novo stage IV breast carcinoma.

## 2 Case Report

A 66‐year‐old female was referred to the ophthalmology clinic with 2.5 weeks of a “bump” on her right lower eyelid (RLL) with gradually worsening right upper eyelid (RUL) drooping for 7 months. She denied pain, double vision, night sweats, thyroid problems, trauma, fever, chills, or lumps under the arms.

**FIGURE 1:**
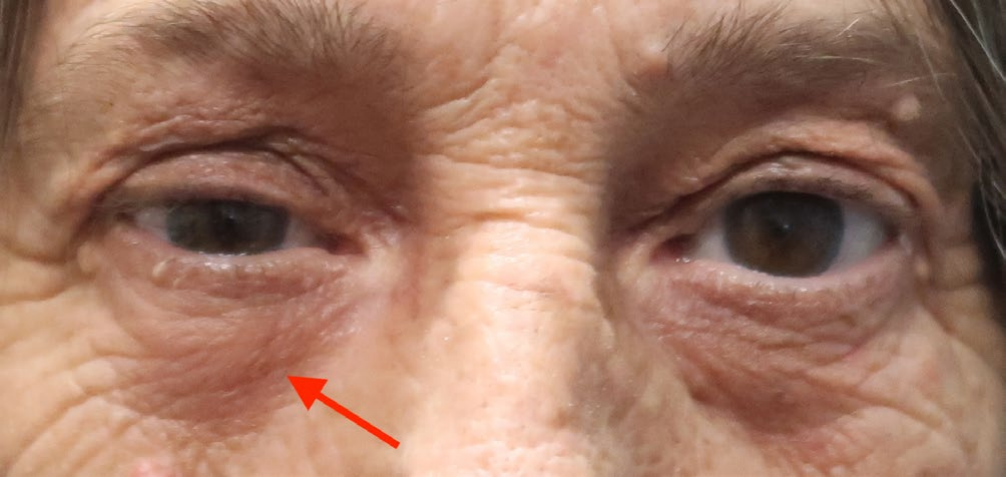
Clinical presentation of eyelid ptosis and nodular growth of the right lower eyelid denoted by the red arrow.

**FIGURE 2:**
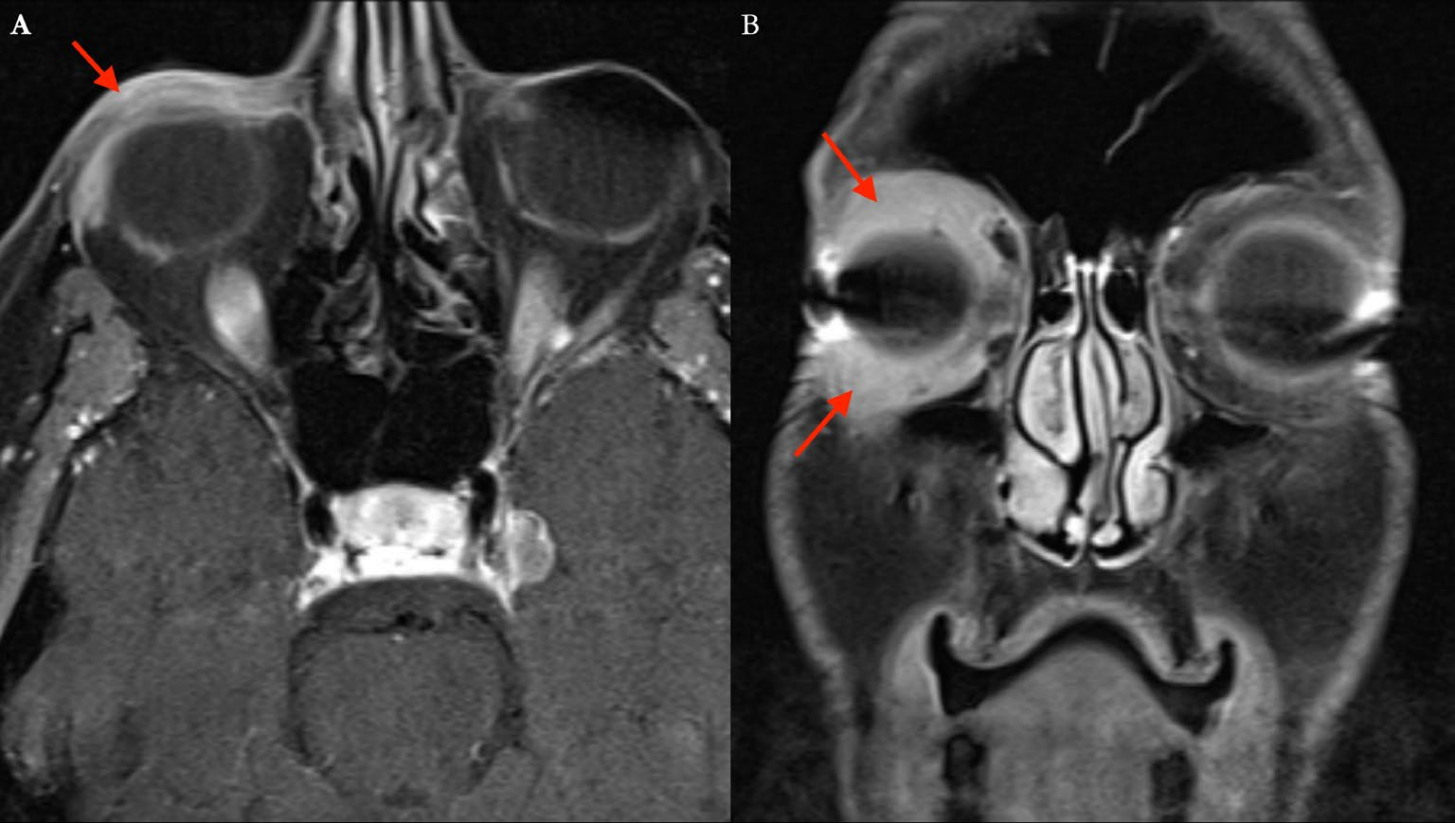
T1‐weighted, fat‐suppressed magnetic resonance images, axial (A) and coronal (B), demonstrate post‐contrast enhancing soft tissue thickening, involving the right upper and lower eyelids denoted by the red arrows.

On physical examination, mild nontender lichenification of the skin and induration of the anterior orbital soft tissue were present along the RUL and RLL (Figure 1). The remainder of the ophthalmic examination was intact with no vision loss, dysmotility, or diplopia. Subsequent MRI of the orbits revealed abnormally enhancing pre‐septal soft tissue superior and inferior to the globe, involving the lids without post‐septal extension thereby reducing suspicion for post‐septal orbital cellulitis and intracranial extension (Figure 2). MRI enhancement was also useful for assessing inflammation of intraocular muscles, thyroid eye disease, and any other intracranial pathologies that could have contributed to the patient's presentation.

**FIGURE 3:**
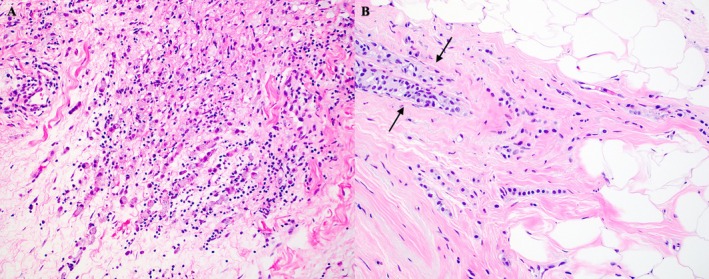
H&E of right lower eyelid biopsy showing cords and strands of malignant cells in linear profile with signet ring cell forms (200×). H&E of right breast biopsy showing breast carcinoma with both lobular and ductal features. Between the arrows, the ductal pattern seen might represent cancerization of ducts or a component of ductal breast adenocarcinoma in addition to the pattern usually seen with lobular carcinoma (200×).

**FIGURE 4:**
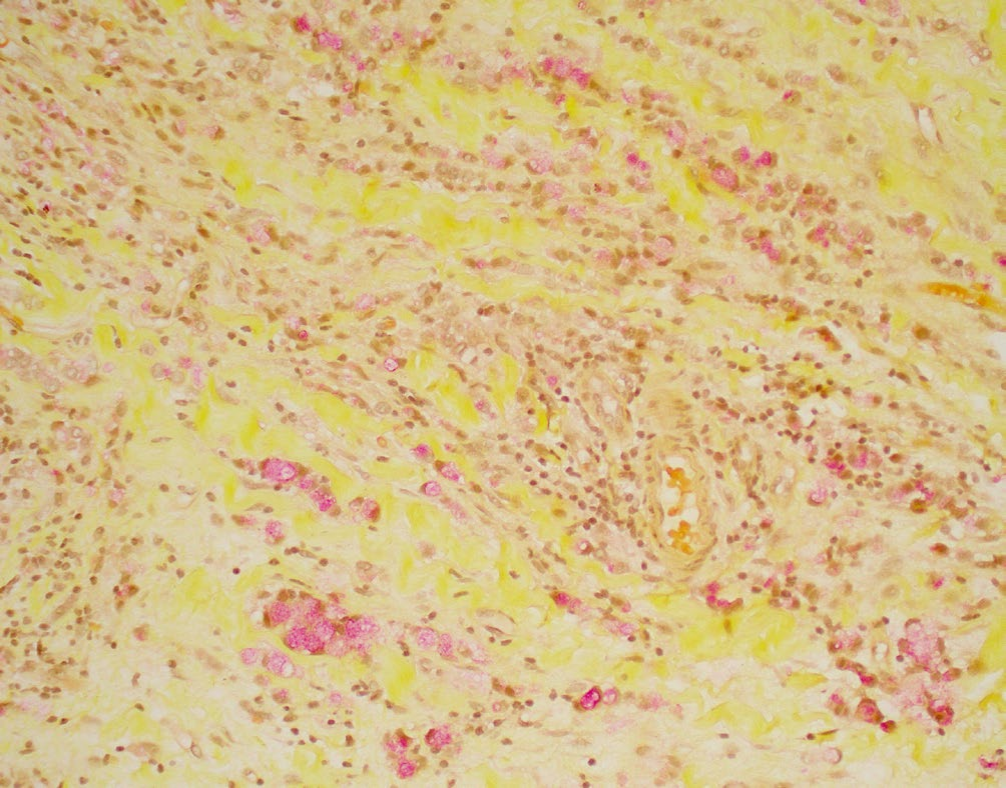
Mucicarmine showing mucin (magenta) within the cytoplasm of the malignant cells, some with signet ring morphology (200×).

**FIGURE 5:**
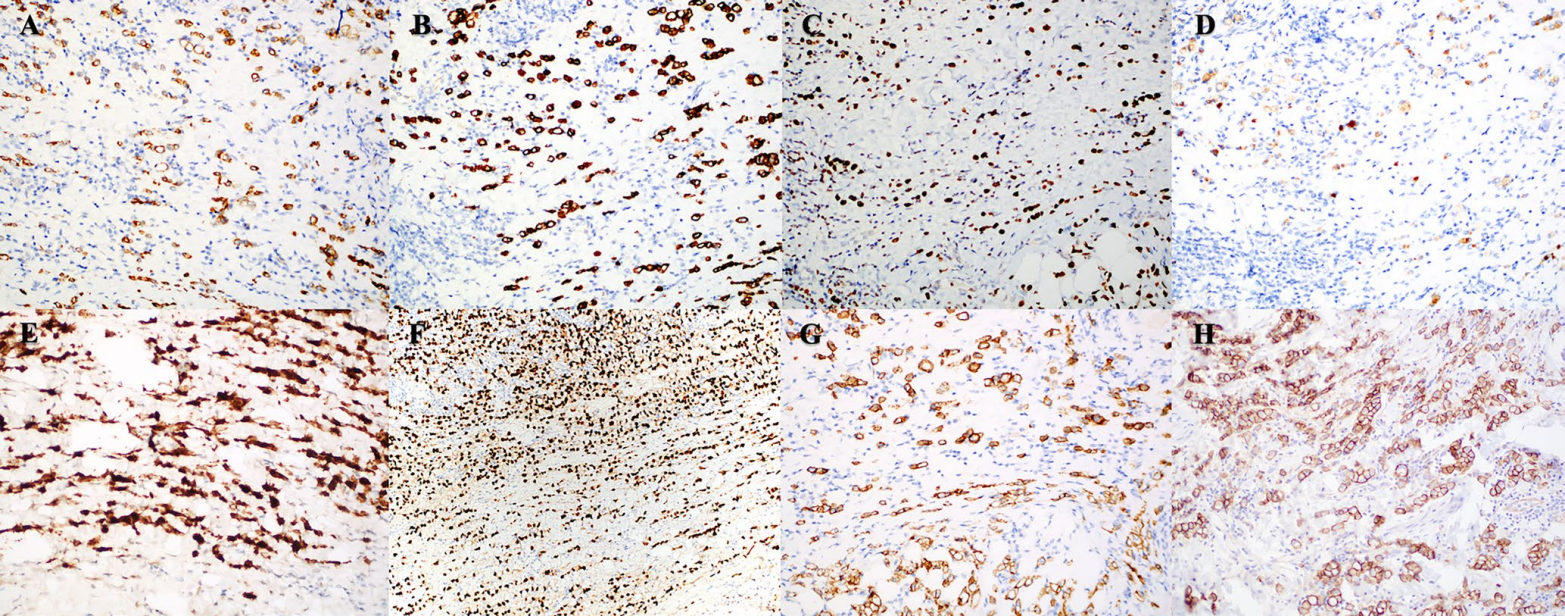
Immunohistochemical profile of the malignant cells on the right lower eyelid biopsy: Keratin CAM5.2 (200×), CK7 (200×), GATA3 (200×), BRST‐2 (200×), Mammaglobin (200×), ER (100×), E‐cadherin (200×), and Membranous uptake of P120 catenin (200×).

RUL and RLL biopsies showed poorly cohesive, infiltrative malignancy growing in linear cords and strands with foci of signet ring cell forms (Figure 3A), positive on a mucicarmine stained tissue section (Figure 4). Immunohistochemical analyses showed positivity for keratin CAM5.2, CK7, GATA3, BRST‐2, mammaglobin, ER, E‐cadherin, and membranous p120‐catenin (Figure 5A–H, respectively) and negativity for CD45, CDX‐2, TTF‐1, melan‐A, and S100. Overall, the constellation of microscopic features and immunohistochemical profile best supported the diagnosis of breast carcinoma, with ILC and IDC highest on the differential. Poorly cohesive malignancies growing in cords and strands with an overall linear architecture and foci of signet ring cells were considered including lymphoma, tubular gastrointestinal tract carcinomas, mucinous carcinoma of the skin, eccrine sweat gland carcinoma and salivary gland carcinoma.

**FIGURE 6:**
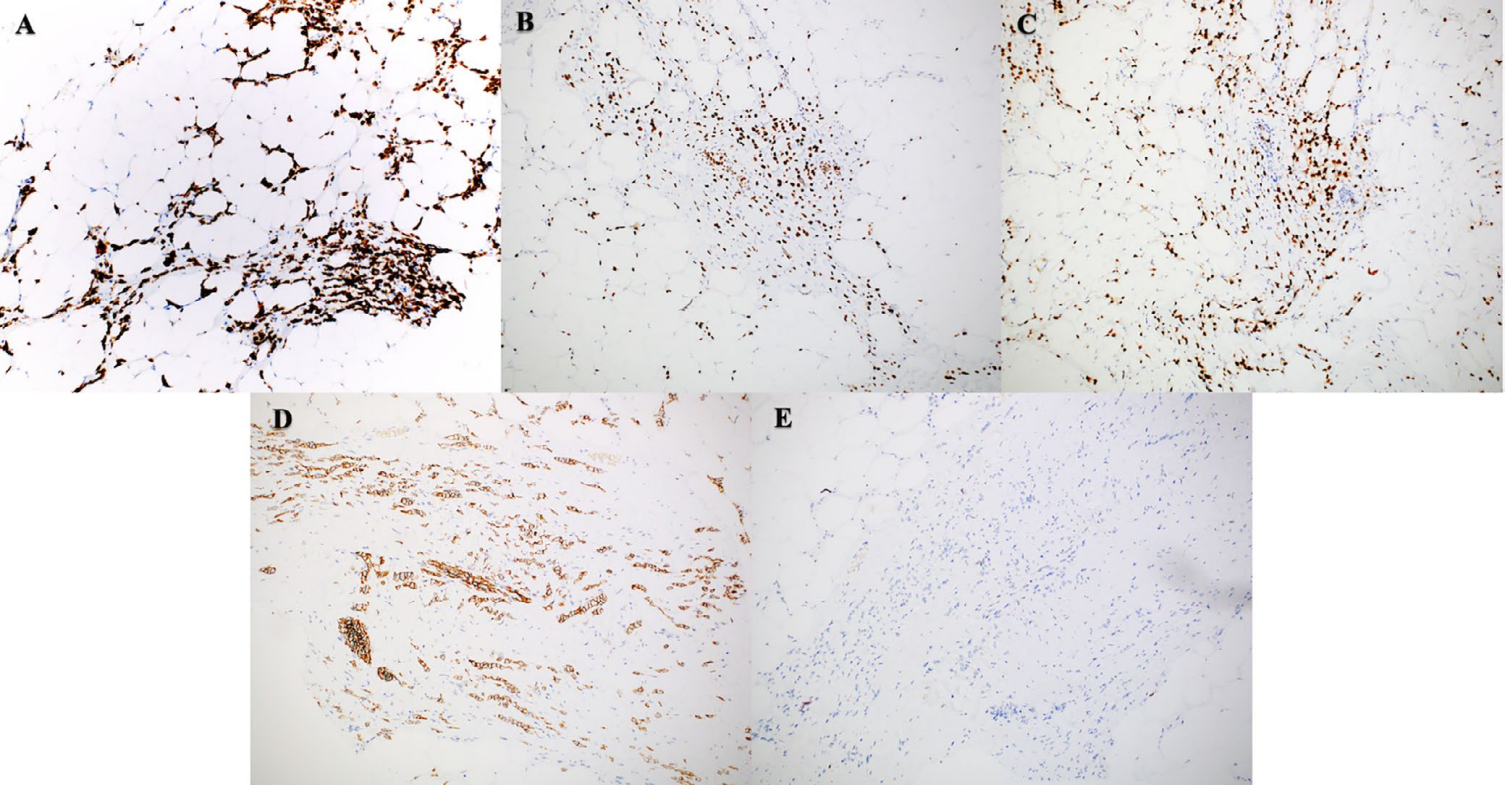
Immunohistochemical profile of malignant cells found on the right breast biopsy (please compare to the eyelid biopsy—Figure 4): CK7 (100×), GATA3 (100×), ER (100×), E‐cadherin (100×) positive, and PR negative (100×).

The patient was referred to oncology, where a palpable right breast mass was found on exam. No salivary gland masses were noted. Estrogen receptor PET scan showed disease in her right breast, right axilla, left cervical nodes, calvarium, and right periocular area. Biopsy of the breast lesion showed breast carcinoma with both lobular and ductal features (Figure 3B). Immunohistochemical studies showed the carcinoma to be CK7, GATA3, ER, and E‐cadherin positive but PR negative (Figure 6A–E, respectively). Furthermore, the biopsy was HER2 negative as well as Ki‐67 positive in 5%–10% of analyzed nuclei, indicating a low proliferative rate.

The patient was diagnosed with stage IV breast cancer and treated with exemestane and CDK4/6 inhibitors. PET imaging a year later showed resolution of previously seen uptake without any new metastatic disease, indicating radiographic remission of the periocular disease. Bone and CT scans the following year showed disease progression, revealing diffuse liver, spine, rib, right pelvis, right lung, and left adrenal gland metastases. 2.5 years after the initial eyelid biopsy, a liver biopsy was performed due to the progression of metastatic lesions, which showed poorly formed groups and single‐file patterning. Immunohistochemistry exhibited positivity for CK7, GATA‐3, mammaglobin, and BRST‐2, which was consistent with metastatic breast carcinoma. E‐cadherin showed retained membranous staining, supporting a ductal immunophenotype. Maintenance exemestane was switched to an estrogen receptor antagonist. CDK4/6 inhibitors were restarted, and bisphosphonate was started for the bone metastases.

## 3 Discussion

Orbital metastases account for 1%–13% of all orbital neoplasms, with breast, melanoma, and prostate representing the most common primary tumors, respectively [11]; most orbital metastases occur in patients with a known primary [12]. The histopathologic differential diagnosis of a poorly cohesive malignancy growing in cords and strands with an overall linear architecture profile and foci of signet ring cells includes the following: lymphoma with signet ring cell morphology, tubular gastrointestinal tract carcinomas with poorly cohesive features and signet ring cells, mucinous carcinoma of the skin, eccrine sweat gland carcinoma, salivary gland carcinoma, primary signet ring cell/histiocytoid carcinoma of the eyelid, high‐grade goblet cell carcinoma of possible appendiceal origin, and breast carcinoma [13, 14, 15, 16].

Many of the primary considerations in the histologic differential diagnosis found in the carcinoma from the eyelid can have similar immunohistochemical profiles. For example, primary breast carcinoma can have neuroendocrine differentiation [17]. Thus, if synaptophysin or chromogranin had been positive, a metastatic breast carcinoma to the eyelid would not have been completely excluded. In contrast, the negative neuroendocrine stains used to initially screen this carcinoma found in the eyelid were negative, making a carcinoma with neuroendocrine differentiation less likely, such as small cell carcinoma, high‐grade goblet cell carcinoma, or carcinoma arising out of a primary neuroendocrine sweat gland carcinoma. Negative CDX‐2 suggested against carcinoma with intestinal differentiation. Lymphoma was excluded due to negativity for CD45. High‐grade neuroendocrine neoplasm, such as high‐grade goblet cell adenocarcinoma, was excluded by negative synaptophysin and chromogranin. Melanoma was ruled out by negative S100 and melan‐A. Negativity for TTF‐1 did not support a lung or thyroid origin. Primary apocrine adenocarcinoma can have similar immunostaining as breast carcinoma, with both having positivity for ER, CK7, mammaglobin, and periodic‐acid Schiff with diastase resistance. However, primary apocrine adenocarcinoma is positive for cytoplasmic iron and negative for adipophilin [18, 19, 20, 21]. Although these stains were not performed for this case patient, primary apocrine carcinoma is extremely rare and, in the setting of a breast mass with similar findings at the time of the eyelid lesion, makes breast carcinoma the most likely diagnosis. Likewise, although some salivary carcinomas and primary signet ring cell carcinoma can have immunoprofiles identical to breast carcinoma, breast carcinoma is more common, and metastases to the eyelids have been previously reported, usually in the context of known prior breast carcinoma [14, 16]. In this case, primary breast carcinoma was confirmed based on physical examination and radiologic studies, with correlation to the metastatic carcinoma found on the eyelid biopsies with positivity for keratin CAM5.2, CK7, GATA3, BRST‐2, mammaglobin, ER, E‐cadherin, and membranous p120‐catenin.

Identification of the breast carcinoma subtype proved diagnostically challenging. Despite the lobular features on microscopy, E‐cadherin expression did not exclude ILC as up to 15% have been observed to have aberrant expression of this glycoprotein [22, 23]. To further delineate its immunophenotype, the eyelid biopsy was stained with p120‐catenin, displaying membranous uptake. In contrast, ILC typically presents with cytoplasmic localization of p120‐catenin [24]. Despite ambiguous histological features of the biopsies, the immunohistochemical findings of E‐cadherin positivity and membranous p120‐catenin provided greater compelling evidence for IDC masquerading as ILC. Prior reports of IDC presenting with a signet ring morphology and mimicking ILC with a pseudo single‐file growth pattern have been documented [24].

Breast carcinoma is the most common origin of orbital metastases, which has been postulated to be due to an affinity for estrogen production by orbital tissues [25, 26]. Eyelid involvement was the initial presentation of this patient's breast cancer. Isolated metastasis to the eyelid of any distant primary cancer is unusual– the most common being cutaneous melanoma [14, 27]. This case highlights the variability in cancer cell behavior and underscores the critical importance of maintaining a broad differential. While metastases of distant primary cancers to the eyelid are rare, a high level of suspicion in any patient with atypical eyelid lichenification will help assure timely diagnosis and management.

## Author Contributions

**Grace L. Casado:** writing – original draft, writing – review and editing, validation, resources, conceptualization. **Eileen Xu:** writing – original draft, writing – review and editing, validation, resources, conceptualization. **Maedot A. Haymete:** writing – review and editing. **Nicholas A. Ramey:** writing – review and editing, validation, resources, data curation. **Douglas J. Grider:** writing – review and editing, validation, resources, conceptualization, visualization, data curation, supervision, project administration.

## Ethics Statement

This case report involved a human subject with assessment, surgical pathology work‐up and treatment usual to routine care. The patient provided Dr. Nicholas Ramey with permission for this case report.

## Conflicts of Interest

Douglas J. Grider, M.D. participated on an Advisory Board for Castle Biosciences on the subject of melanoma prognostic ancillary testing; all other authors declare no conflicts of interest.

## Data Availability

The data that support the findings of this study are available on request from the corresponding author. The data are not publicly available due to privacy or ethical restrictions.
